# McConnell’s sign; a distinctive echocardiographic finding for diagnosing acute pulmonary embolism in emergency department

**DOI:** 10.1186/2036-7902-7-S1-A20

**Published:** 2015-03-09

**Authors:** Seong Beom Oh, Seung Jae Bang, Min Jeong Kim

**Affiliations:** Department of Emergency Medicine, College of Medicine, Dankook University, Cheonan, Republic of Korea

**Keywords:** Emergency Department, Pulmonary Artery, Acute Respiratory Distress Syndrome, Main Pulmonary Artery, Segmental Artery

## Background

McConnell’s sign, that is regional RV dysfunction, with akinesia of the mid free wall but normal motion at the apex is a distinct echocardiographic finding described in patients with acute pulmonary embolism.

## Objective

The aim of this prospective study was to analyze clinical usefulness of McConnell’s sign for diagnosing acute pulmonary embolism before computed tomography scan in emergency department.

## Patients and methods

From June 2013 through August 2014, 15 patients who were observed McConnell’s sign from bedside echocardiography performed by emergency medicine specialist were enrolled in this study. Those whose final diagnosis was acute pulmonary embolism were classified into three groups such as main, lobar and segmental pulmonary artery thrombus group according to the thrombus location based on the CT scan. Demographic and various clinical data were recorded and analyzed.

## Results

Average age was 64.7±15.6 and female was 9(60%). In 15, final diagnosis of two subjects was not acute pulmonary embolism. One was acute respiratory distress syndrome and the other was inferior wall STEMI with RV infarction. The rest of the subjects(13 of 15, 86.7%) were diagnosed acute pulmonary embolism with CT scan. In these subjects, 12(92.3%) were identified as huge thrombus in main pulmonary artery and the other in lobar pulmonary artery. There was no subject whose thrombus was identified in segmental pulmonary artery.

## Conclusion

Regional RV dysfunction known as McConnell’s sign was very distinctive echocardiographic finding for diagnosing acute pulmonary embolism in emergency department. In addition, identification of this sign strongly suggests massive thrombus burden such as main pulmonary artery thrombus rather than lobar or segmental artery.
